# Premovement activity in the mesocortical system links peak force but not initiation of force generation under incentive motivation

**DOI:** 10.1093/cercor/bhad376

**Published:** 2023-10-09

**Authors:** Sho K Sugawara, Tetsuya Yamamoto, Yoshihisa Nakayama, Yuki H Hamano, Masaki Fukunaga, Norihiro Sadato, Yukio Nishimura

**Affiliations:** Neural Prosthetics Project, Tokyo Metropolitan Institute of Medical Science, Setagaya, Tokyo 156-8506, Japan; Section of Brain Function Information, National Institute for Physiological Sciences, Okazaki, Aichi 444-8585, Japan; The Graduate University for Advanced Studies, SOKENDAI, Hayama, Kanagawa 340-0193, Japan; Section of Brain Function Information, National Institute for Physiological Sciences, Okazaki, Aichi 444-8585, Japan; Neural Prosthetics Project, Tokyo Metropolitan Institute of Medical Science, Setagaya, Tokyo 156-8506, Japan; Section of Brain Function Information, National Institute for Physiological Sciences, Okazaki, Aichi 444-8585, Japan; Section of Brain Function Information, National Institute for Physiological Sciences, Okazaki, Aichi 444-8585, Japan; The Graduate University for Advanced Studies, SOKENDAI, Hayama, Kanagawa 340-0193, Japan; Section of Brain Function Information, National Institute for Physiological Sciences, Okazaki, Aichi 444-8585, Japan; The Graduate University for Advanced Studies, SOKENDAI, Hayama, Kanagawa 340-0193, Japan; Research Organization of Science and Technology, Ritsumeikan University, Kusatsu, Shiga 525-8577, Japan; Neural Prosthetics Project, Tokyo Metropolitan Institute of Medical Science, Setagaya, Tokyo 156-8506, Japan

**Keywords:** ventral midbrain, motor cortex, reaction time, force, reward

## Abstract

Motivation facilitates motor performance; however, the neural substrates of the psychological effects on motor performance remain unclear. We conducted a functional magnetic resonance imaging experiment while human subjects performed a ready-set-go task with monetary incentives. Although subjects were only motivated to respond quickly, increasing the incentives improved not only reaction time but also peak grip force. However, the trial-by-trial correlation between reaction time and peak grip force was weak. Extensive areas in the mesocortical system, including the ventral midbrain (VM) and cortical motor-related areas, exhibited motivation-dependent activity in the premovement “Ready” period when the anticipated monetary reward was displayed. This premovement activity in the mesocortical system correlated only with subsequent peak grip force, whereas the activity in motor-related areas alone was associated with subsequent reaction time and peak grip force. These findings suggest that the mesocortical system linking the VM and motor-related regions plays a role in controlling the peak of force generation indirectly associated with incentives but not the initiation of force generation.

## Introduction

Empirical evidence indicates that motivation enhances motor performance, demonstrated by outcomes such as improvements in reaction time (Hollerman et al. 1998; Hassani et al. 2001; Adcock et al. 2006; Mir et al. 2011) and force exertion (Pessiglione et al. 2007; Schmidt et al. 2012). Thus, motivation and the motor systems appear to be closely related in the central nervous system. Motivation-driven behaviors are associated with the activation of dopaminergic (DA) neurons in the ventral midbrain (VM), including the ventral tegmental area and substantia nigra pars compacta. Most DA neurons show phasic activation following reward-predicting stimuli (Berridge and Robinson 1998; Schultz 1998; Matsumoto and Hikosaka 2009; Haber and Knutson 2010). Specifically, shorter reaction times are considered a proxy for greater motivation (Hollerman et al. 1998; Hassani et al. 2001; Adcock et al. 2006; Mir et al. 2011), but there is no direct evidence of a relationship between the activation of the VM and subsequent reaction times. Human neuroimaging studies have also demonstrated that the mesolimbic system, including the VM, represents expected rewards (Knutson et al. 2003, 2005; Wittmann et al. 2005; Carter et al. 2009; Liu et al. 2011).

Descending commands for controlling voluntary limb movements are generated in the motor cortex and activate spinal motoneurons and interneurons. Numerous studies have shown that neural activity in the motor cortex represents various motor parameters, such as initiation (Nashef et al. 2018) and generated force (Evarts et al. 1983; Riehle and Requin 1993). Human neuroimaging studies have also demonstrated force-related activity in motor-related areas, including the primary motor cortex (M1) and supplementary motor area (SMA) (Dettmers et al. 1995; Cramer et al. 2002; Kuhtz-Buschbeck et al. 2008). Furthermore, reward anticipation increases the excitability of corticospinal tracts from the M1 (Kapogiannis et al. 2008; Thabit et al. 2011; Klein et al. 2012; Galaro et al. 2019). Thus, reward-predicting stimuli influence the activity of motor-related cortical regions, which play an important role in generating motor output and directly innervating the spinal cord. However, it is unclear whether the neural activity associated with reward anticipation influences subsequent motor performance.

To investigate the neural substrates linking motivational incentives and subsequent motor performances, we conducted functional magnetic resonance imaging (fMRI) of human subjects. We measured task-related brain activity during a ready-set-go task with monetary incentives and investigated the brain regions in which premovement activity was correlated with subsequent motor parameters (reaction time and peak grip force).

## Materials and methods

### Subjects

A total of 28 healthy volunteers (13 males and 15 females, mean age ± standard deviation = 22.32 ± 2.63 years) participated in this study. One male subject did not complete all tasks due to claustrophobia and was excluded from further analysis. None of the subjects had a history of neurological or psychiatric disorders. All subjects had normal or corrected normal vision and were right-handed according to the Edinburgh Handedness Inventory (Oldfield 1971). Written informed consent was obtained from all subjects before participation in the experiments, and the study was conducted in accordance with the Declaration of Helsinki. This study was approved by the Ethics Committee of the National Institute for Physiological Sciences, Japan, and the Tokyo Metropolitan Institute of Medical Science, Japan.

### Experimental design

During scanning, subjects lay in the MRI scanner with their heads immobilized by an elastic band and sponge cushions and with their ears plugged. Stimulus presentation and response collection were conducted using Presentation software (Neurobehavioral Systems) implemented on a personal computer (dc7900; Hewlett-Packard). An LCD projector (CP-SX12000; Hitachi) located outside and behind the scanner projected the stimuli through a waveguide to a translucent screen, which the subjects viewed via a mirror placed in the MRI scanner. The spatial resolution of the projector was 1,280 × 960 pixels, with a 60-Hz refresh rate. The distance between the screen and the subjects’ eyes was ~175 cm, and the visual angle was 13.8° (horizontal) × 10.4° (vertical). The subjects held an MRI-safe optical grip force device (Current Design) that was able to measure up to 500 N with their right hand during the scans.

While participants were in the MRI scanner, we first measured the subjects’ maximum grip force three times to determine their individual thresholds. In this task, the subjects were instructed to squeeze the device as strongly as possible while the instruction message “Squeeze” was displayed in Japanese for 5 s and to stop squeezing while the instruction message “Rest” was displayed in Japanese for 5 s. We recorded the averaged maximum force power across the three measurements as the maximum voluntary contraction (MVC).

**Fig. 1:**
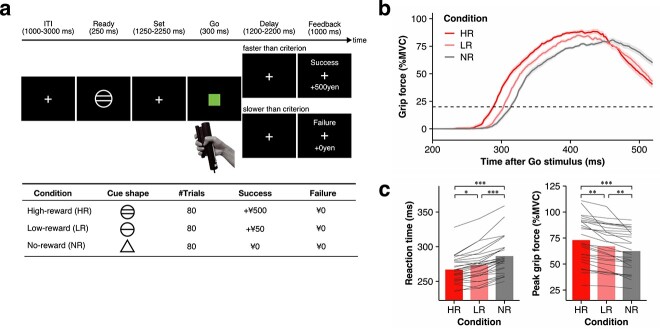
Incentive ready-set-go task. (a) Trial procedure for the incentive ready-set-go task. In each trial, the subject was asked to prepare to respond when the “ready” cue was presented and to respond to the appearance of the “go” stimulus as quickly as possible. To manipulate motivation, the task was conducted under three conditions corresponding to different monetary reward amounts for fast responses: ¥500 (HR condition), ¥50 (LR condition), and ¥0 (NR condition). ITI, intertrial interval. (b) Mean grip force trajectory in each condition. The grip force trajectories obtained from a representative subject (M.I.) in the three conditions: HR (red), LR (light red), and NR (gray). Lines and shaded areas represent the mean grip force and standard error of the mean, respectively. MVC, maximum voluntary contraction. (c) Motor performance of all subjects. Reaction times (left) significantly decreased in the following order: HR < LR < NG. In addition, peak grip force was significantly greater in the following order: HR > LR > NG. ^***^p < 0.001, ^**^p < 0.01, and ^*^p < 0.05.

Then, the subjects performed a simple ready-set-go task (Fig. 1a) that was modified from a monetary delay incentive task (Knutson et al. 2000, 2001, 2003). In each trial, an open white triangle appeared for 300 ms as a “Ready” cue. During the “Set” period of 1,250–2,250 ms, a white-colored crosshair was presented. Then, when a solid green square was displayed for 300 ms, the subjects were instructed to squeeze the grip force device as quickly as possible. After a variable interval of delay (1,200–2,200 ms), a feedback message was displayed on the screen for 1,000 ms. If a subject responded faster than the reaction-time threshold explained below, the word “Success” was displayed. If a subject responded slower than the reaction-time threshold, the word “Failure” was displayed. Intertrial intervals were varied according to the duration of the delay periods (1,000–3,000 ms). First, the subjects performed two short practice sessions to familiarize themselves with the task and to measure their baseline reaction time. Each practice session consisted of 10 trials. The reaction-time threshold was calculated as the median reaction time during the second practice session. Next, subjects performed the simple ready-set-go task for two sessions. The reaction-time threshold was updated to the median pooled reaction time from the simple ready-set-go task.

After the simple ready-set-go task, subjects performed the incentive ready-set-go task for eight sessions. Although the trial procedure was identical to that of the simple ready-set-go task, the incentive ready-set-go task had three conditions corresponding to three different amounts of monetary reward: high reward (HR), low reward (LR), and no reward (NR) conditions. These conditions were denoted by an open white circle with two horizontal lines, an open white circle with one horizontal line, and an open white triangle, respectively. In the HR and LR conditions, subjects received 500 and 50 Japanese yen per trial, respectively, if their response was faster than the reaction-time threshold. The determination of whether the response was fast or not was dependent only on reaction time and was unrelated to the peak grip force. Each session included 30 task trials (10 trials per condition) and five null trials, in which only the fixation cross was displayed for 7 s. The order of conditions was pseudorandomized and was identical across all subjects.

Before entering the MRI scanner, the subjects were instructed that they could receive the amount of money earned in any session as an additional reward. After completing eight sessions of the incentive ready-set-go task, subjects underwent a lottery task to determine which session’s rewards were awarded as an additional prize. All subjects were paid a fixed amount for their participation and the additional prize at the end of the experiment.

### MRI data acquisition

A 3.0 T scanner (Verio; Siemens Healthcare GmbH) was used for the fMRI study. fMRI was performed using multiband echo-planar imaging (EPI) (Moeller et al. 2010; multiband factor = 6, repetition time [TR] = 1,000 ms, echo time [TE] = 35 ms, field of view [FOV] = 192 × 192 mm^2^, flip angle = 65°, matrix size = 96 × 96, 64 slices, slice thickness = 2.0 mm, and phase partial Fourier = 6/8). The number of scans was 275 per run. To correct susceptibility-induced distortion by using the “Topup” toolbox in FSL (Andersson et al. 2003), two spin–echo EPI sequences with reversed-phase encoding directions were performed (TR = 7560 ms, TE = 64 ms, FOV = 192 × 192 mm^2^, FA = 90°/180°, matrix size = 96 × 96, 60 slices, slice thickness = 2 mm, and phase partial Fourier = 6/8).

To apply the Human Connectome Project (HCP) pipelines to our data, a series of structural images from all subjects were obtained on a day other than the testing day. Two separate sets of T1- and T2-weighted images were acquired using a 3D magnetization-prepared rapid-acquisition gradient echo (MPRAGE) sequence (Mugler and Brookeman 1990; TR = 2,400 ms, TE = 2.24 ms, FOV = 256 × 240 mm^2^, flip angle = 8°, matrix size = 320 × 320, slice thickness = 0.8 mm, 224 sagittal slices, and GRAPPA = 2) and a variable flip angle turbo spin–echo sequence (Siemens SPACE; Mugler et al. 2000; TR = 3,200 ms, TE = 560 ms, FOV = 256 × 240 mm^2^, matrix size = 320 × 320, slice thickness = 0.8 mm, 224 sagittal slices, and GRAPPA = 2), respectively. Similar to the fMRI session, two spin–echo EPI sequences with reversed-phase encoding directions were performed (TR = 7,700, TE = 60 ms, FOV = 208 × 208 mm^2^, FA = 78°/160°, matrix size = 104 × 104, 72 slices, slice thickness = 2 mm, and phase partial Fourier = 6/8).

### MRI data preprocessing

MRI preprocessing was performed using the HCP minimal processing pipelines (Glasser et al. 2013; Yamamoto et al. 2021), although only the volume-based components were used. HCP-style preprocessing consisted of structural and functional pipelines.

The structural pipeline was applied to the T1- and T2-weighted images. First, image distortions resulting from gradient nonlinearity were corrected. After the brain region was extracted, susceptibility-induced distortions were corrected using two spin–echo EPI sequences with opposite phase-encoding directions and the Topup toolbox (Andersson et al. 2003). Undistorted T1- and T2-weighted images were registered with BBR cross-modal registration. As the intensity of the T1- and T2-weighted images still had biases, bias-field correction was applied to the undistorted images. Finally, the nonlinear registration warp field from native space to the Montreal Neurological Institute (MNI) template space was estimated, and this nonlinear registration was applied to the T1- and T2-weighted images.

The functional pipeline also started with correction for gradient nonlinearity-induced distortion. To correct for head motion, echo-planar (EP) images were registered to a single-band reference EP image, which was scanned at the beginning of each run, by estimating six parameters of rigid-body transformation from each EP image to the single-band reference EP image. In this study, structural and functional MRI sessions were conducted on different days. Thus, a session-specific field map was calculated from two spin–echo EP images obtained in the fMRI session. Using this field map, susceptibility-induced distortion correction was applied to motion-corrected EP images with the Topup toolbox. The transformation matrix from the single-band reference EPI to the T1-weighted image was estimated by the cross-modal boundary-based registration (BBR) method. Then, this BBR parameter was applied to undistorted EP images to register all EP images into T1-weighted images. The resultant EP images were transformed to MNI template space using the T1-weighted-to-MNI parameters estimated in the structural pipelines with bias-field correction applied. Finally, the image intensities of EP images were normalized to the 4D whole-brain mean of 10,000. To smooth EP images, the normalized functional images were filtered using a 4-mm full width at half maximum (FWHM) Gaussian kernel in the *x*, *y*, and *z* axes. To distinguish the noise components from the fMRI time series and to remove the identified noise components, the “multirun” version of FSL FIX (Griffanti et al. 2014; Salimi-Khorshidi et al. 2014) was applied to the concatenated time series across all runs. First, by using spatial independent component analysis (ICA) with FSL Melodic, the time-series data were divided into several components. The estimated ICA components were classified automatically into “signal” and “noise” components with predefined classification parameters trained for the HCP dataset. Since our MRI environment had different scanner hardware and scanning parameters than the HCP environment, the labels resulting from automatic classification were manually reviewed according to the proposed guidelines (Griffanti et al. 2017).

### Statistical analysis

#### Behavioral data

The grip force trajectory was recorded in each trial. Using these trajectories, the reaction time and maximum force were estimated in a trial-by-trial manner. Reaction time was defined as the time at which the force reached 20% of the MVC (hereafter, 20% MVC). Maximum force was defined as the maximum value of force after the presentation of the target stimulus.

According to performance, trials were divided into three types: correct, omission, and false start. Trials in which grip force power did not reach 20% MVC from the onset of the Go stimulus to the onset of feedback were defined as “omission” trials. Trials in which grip force power reached 5% MVC before the onset of the Go stimulus were defined as “false-start” trials. The other trials were defined as “correct” trials. Individual motor performance was calculated only for the correct trials. For individual motor performance, median reaction time and peak grip force were calculated in each incentive condition. In addition, the ratio of correct trials (the correct rate) was calculated as well as the number of omission and false-start trials in each incentive condition.

The Shapiro–Wilk normality test showed that behavioral performance variables were not normally distributed (reaction time: *p* = 4.58 × 10^−4^; peak grip force: *p* = 0.045). Thus, the Friedman test was conducted to examine response time and maximum force across incentive conditions (HR/LR/NR) to test the effect of the incentive cue on behavioral performance. Then, post hoc comparisons were performed by using Durbin-Conover tests with Bonferroni correction. In addition, nonparametric correlation analyses with Kendall’s rank correlation were conducted between peak grip force and reaction time across all trials and in each condition to investigate the within-subject relationship between the two types of motor performance. To determine whether the median correlation coefficient significantly differed from zero, a one-sample Wilcoxon signed-rank test was conducted. The significance level was set to 0.05 for all behavioral analyses. These statistical tests were performed using R version 4.2.2 statistical software (http://cran.us.r-project.org).

#### Task-related activation

After HCP-style preprocessing, task-related activity was analyzed with Statistical Parametric Mapping software (SPM12; Wellcome Trust Center for Neuroimaging) in MATLAB 2018a. The first five volumes of each fMRI run were discarded because the signal was not stable. Statistical analysis of the fMRI data was conducted at two levels. At the first level, a general linear model (GLM) was fitted to the fMRI data for each subject (Friston et al. 1994; Worsley and Friston 1995).

Two types of GLMs were applied to identify the brain regions related to the effect of the incentive conditions and actual motor performance during the Ready phase. To determine the brain regions representing the effect of the incentive condition on preparatory activity, GLM1 modeled the three different incentive conditions (HR, LR, and NR) as separate regressors as well as the Go stimulus and feedback. Thus, GLM1 contained five regressors: three for the three different incentive conditions indicated by the Ready cue modeled with a stick function, one for the Go stimulus modeled with a stick function, and one for Feedback modeled with a boxcar function (fixed duration of 1,000 ms). To determine the brain regions correlated with motor performance after the Go stimulus, GLM2 contained three event-related regressors (i.e. Ready, Go, and Feedback) without considering conditions and modeled two additional ready-related regressors parametrically modulated with trial-by-trial peak grip force and reaction time.

The aforementioned GLMs modeled the regressors corresponding to the correct trials. For all GLMs, if omission and/or false-start trials were present, an additional three regressors modeling the three trial types (correct, omission, or false start) were included. For all GLMs, all regressors were convolved with the canonical hemodynamic response function. In addition, to address the effect of head motion, 24 nuisance regressors modeling head motion (derived from six motion parameters, their first temporal derivatives, and the squares of these 12 resulting regressors) were included in each GLM (Satterthwaite et al. 2013; Griffanti et al. 2014). The time series for each voxel was high-pass filtered at 1/128 Hz. For advanced rapid sampling techniques, such as the multiband gradient–echo EPI sequence used in this study, a first-order autoregressive model does not sufficiently capture temporal correlations in time series with higher sampling rates (Bollmann et al. 2018). Thus, the “FAST” model implemented in SPM12 was used to address the temporal correlations in time series with higher sampling rates (Corbin et al. 2018). Then, to calculate the estimated parameters, a least-squares estimation was performed on the high-pass filtered and prewhitened data.

GLM1 estimated three linear contrasts. First, the mean ready-related activity across the three incentive conditions was compared with the activity during the implicit rest phases (i.e. null trials) to illustrate mean activation related to motor preparation. Second, to determine the activation related to the anticipated monetary reward, the mean preparatory activity in the HR and LR conditions was compared to that in the NR condition. Third, to determine the brain regions in which the preparatory activity depended on the amount of monetary reward, preparatory activity in the HR condition was compared with that in the LR condition. GLM2 was used to determine the brain regions in which preparatory activity was correlated with future motor performance; thus, two parametric modulation contrasts were estimated (modulation with peak grip force and reaction time).

These linear contrasts from first-level analyses were then incorporated in the group-level random-effect analysis using one-sample *t-*tests. The resulting set of voxel values for each contrast constituted SPM{*t*}, which was transformed into normal distribution units (SPM{*z*}). For whole-brain analysis, the threshold for SPM{*z*} was set to *Z* = 3.09 (equivalent to *p* < 0.001 uncorrected). The statistical threshold for the spatial extent test on the clusters was set at *p*< 0.05 and corrected for multiple comparisons over the whole-brain mask (Friston et al. 1996). Typically, the MRI signal in ventral brain regions, including the nucleus accumbens and midbrain, is less intense than the signal in lateral cortical brain regions. Thus, the automatically defined brain mask created in the group-level analysis often lacks ventral brain regions. To address this issue, an explicit brain mask generated by averaging the extracted individual brain mask created in the structural pipeline was used. Moreover, small-volume correction analysis was applied for all GLMs to precisely evaluate the activity in the VM. For small-volume correction, VM and VP masks were generated according to the MRI-based in vivo atlas of human subcortical brain nuclei (Pauli et al. 2018). The VM was defined as the midbrain region including the substantia nigra pars compacta, parabrachial pigmental nucleus, and ventral tegmental area. The statistical threshold for small-volume correction was set at *p* < 0.05 and corrected for multiple comparisons over the VM mask. In addition, the extent threshold was set as 5 and 3 voxels for the VM and VP, respectively.

Brain regions were defined automatically and labeled according to an automated anatomical labeling atlas (Tzourio-Mazoyer et al. 2002; Rolls et al. 2015), a connectivity-based parcellation atlas for the cingulate cortex (Neubert et al. 2015), and a probabilistic in vivo atlas of human subcortical nuclei for the midbrain (Pauli et al. 2018).

**Fig. 2:**
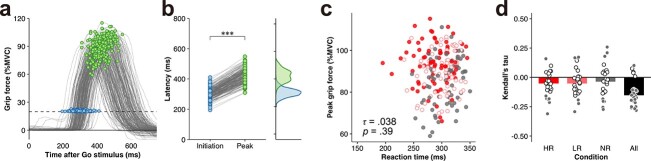
The trial-by-trial relationship between the initiation and the peak of grip force. (a) Trial-by-trial grip force trajectories from a representative subject (M.I.). (b) The latency to the initiation and peak of trial-by-trial responses. Dots represent latencies in each trial. The distribution of the latencies to the initiation and peak are also shown in the right panel. The reaction time (blue) as the latency to initiation was significantly faster than the latency to peak grip force (green). ***p < 0.001. (c) The scatter plot of trial-by-trial reaction time and peak grip force from a representative subject (M.I.). The color of the circles represents the condition: HR (red filled circles), LR (light red open circles), and NR (gray filled circles). (d) Kendall’s correlation coefficients were calculated for each condition and subject. Each circle denotes the correlation coefficient for each subject and condition. The color of the circles indicates significant (gray) and nonsignificant (white) correlations. Bars represent the median correlation coefficient across subjects in each condition: HR (red bars), LR (light red bars), NR (gray bars), and all conditions (black bars). The significance level was set as *p* < 0.05 for correlational analyses.

## Results

### Task performance in the incentive ready-set-go task

The subjects (*n* = 28) performed a ready-set-go task with monetary incentives (Fig. 1a; see Materials and Methods) modified from a monetary incentive delay task (Knutson et al. 2000, 2001, 2003). The average grip force trajectories of a representative subject are shown in Fig. 1b. In the behavioral task, participants were motivated to grip the device as quickly as possible because differing levels of monetary reward according to the incentive condition (HR, LR, and NR) were given for reaction times faster than the threshold. Reaction times were shorter in the following order: HR < LR < NR. These results confirmed that a faster reaction time is a proxy for a higher motivational state. At the group level, reaction time was significantly decreased in the same order (HR < LR < NR) (Fig. 1c; Friedman test, *χ^2^*(2) = 47.19, *p* = 5.67 × 10^−11^; Bonferroni-corrected post hoc pairwise comparisons: HR vs. LR, *p* = 0.038; HR vs. NR, *p* = 3.00 × 10^−8^; LR vs. NR, *p* = 3.00 × 10^−4^).

Unexpectedly, even though the peak grip force was not related to whether participants would receive monetary rewards, peak grip force followed the same pattern (HR > LR > NR) (Fig. 1b). This tendency was robust across participants (Fig. 1c; Friedman test, *χ^2^*(2) = 54.00 *p* = 1.88 × 10^−12^; Bonferroni-corrected post hoc pairwise comparisons: HR vs. LR, *p* = 1.69 × 10^−3^; HR vs. NR, *p* = 4.13 × 10^−9^; LR vs. NR, *p* = 1.69 × 10^−3^). Thus, the peak grip force was indirectly dependent on the motivational state, which varied according to the anticipated monetary reward similar to the reaction time.

As shown above, the behavioral results clearly demonstrated that the anticipation of monetary reward not only improved reaction time but also indirectly improved peak grip force (Fig. 1b and c). Nevertheless, as shown by the trial-by-trial grip force trajectories from a representative subject (Fig. 2a), the reaction times and peak grip forces were variable across trials. Obviously, the latency to peak force was consistently longer than the reaction time (i.e. response initiation) (Fig. 2b; Wilcoxon signed-rank test, *V* = 0, *p* < 10^−16^). At the population level, the latency to initiation, as measured by reaction time (ranging from 240 to 332.5 ms), differed temporally from the latency to peak grip force (ranging from 311 to 457.5 ms) across all participants (Wilcoxon signed-rank test, *V* = 0, *p* < 10^−16^). This temporal difference suggests that the initiation and peak of force generation are controlled in distinct time windows. Furthermore, the correlation between reaction time and peak grip force across trials was not strong (Fig. 2c) and was variable among subjects (Fig. 2d). In the HR condition, the correlations ranged from −0.31 to 0.16 and were significant in only 7 out of 27 subjects (median = −0.053; one-sample Wilcoxon signed-rank test, *V* = 76, *p* = 5.47 × 10^−3^). In the LR condition, the correlations ranged from −0.29 to 0.17 and were significant in only 5 out of 27 subjects (median = −0.052; one-sample Wilcoxon signed-rank test, *V* = 97, *p* = 0.026). In the NR condition, the correlations ranged from −0.27 to 0.26 and were significant in only 9 out of 27 subjects (median = −0.038; one-sample Wilcoxon signed-rank test, *V* = 126, *p* = 0.13). In the data from all trials, 22 out of 27 subjects showed a significant correlation between reaction time and peak grip force (range, −0.28 to 0.10; Fig. 2d). Although statistical analysis supported a significant negative correlation at the group level (one-sample Wilcoxon signed-rank test, *V* = 17, *p* = 3.08 × 10^−6^), the median correlation coefficient across subjects was low (median = −0.15). These results indicated that the trial-by-trial relationship between reaction time and peak grip force is very weak, raising the possibility that the motivational state differentially affects the initiation and peak of force generation through different neural substrates.

### Incentive-related preparatory brain activity

**Fig. 3:**
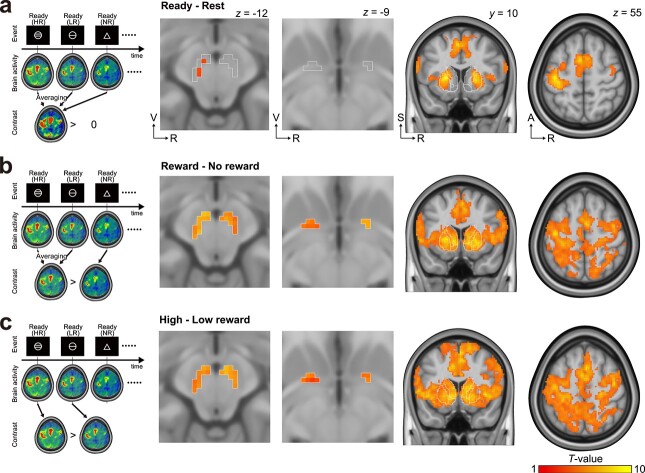
Preparatory activity during the ready period. (a) Mean preparatory activity across all trials. A, anterior; R, right; S, superior; V, ventral. (b) Effect of reward on preparatory activity (i.e. [HR + LR] > NR conditions). (c) the effect of the amount of anticipated monetary reward on preparatory activity (i.e. HR > LR conditions). The first column shows outlines of linear contrasts. The second and third columns show the results from small-volume correction. The regions of interest, which are outlined with white lines, indicate the ventral midbrain (left-most panel; including the bilateral ventral tegmental area, parabrachial pigmented nucleus, and substantia nigra pars compacta) and ventral pallidum (second panel from left), defined by the in vivo atlas of human subcortical brain nuclei (Pauli et al. 2018). The fourth and fifth columns display the results from whole-brain analysis. Outlined regions in the third column represent the nucleus accumbens, caudate, and putamen, defined by the in vivo atlas of human subcortical brain nuclei.

Motor performance was strongly modulated by incentive motivation, which was manipulated by the amount of the anticipated monetary reward (Fig. 1). To identify incentive-related activity, we first analyzed task-related activity during the Ready period because the motivational state was altered by the shape of the Ready cue, which indicated the amount of anticipated monetary reward. Although no significant activation was observed in the nucleus accumbens (NAc) or ventromedial prefrontal cortex (VMPFC) when comparing the Ready-related activity averaged across all conditions with the resting-period activity (second panel from the right in Fig. 3a and Supplementary Table 1), the bilateral NAc (cluster-level *p_FWE_* < 0.05 with a height threshold *p_uncorr._* < 0.001; second panel from the right in Fig. 3b) and VMPFC (Supplementary Table 1) were strongly activated in the anticipated monetary reward conditions (i.e. HR and LR) compared to the no-monetary reward condition (i.e. NR). The degree of activity in these brain regions was modulated according to the amount of anticipated monetary reward (HR > LR; second panel from the right in Fig. 3c). These results are consistent with previous evidence of reward anticipation (Knutson et al. 2003, 2005).

We next examined task-related activity in reward-related subcortical regions during the Ready period. The VM is involved in the processing of reward-related and motivational signals (Berridge and Robinson 1998; Schultz 1998; Knutson et al. 2005; Carter et al. 2009; Matsumoto and Hikosaka 2009; Haber and Knutson 2010). Additionally, the ventral pallidum (VP) is involved in reward-related processing (Pessiglione et al. 2007; Root et al. 2015). Therefore, we focused on these regions. The bilateral VM (*p_SVC_* < 0.05; left-most panel in Fig. 3b and Supplementary Table 2) and VP (*p_SVC_* < 0.05; second panel from the left in Fig. 3b) were strongly activated in the anticipated monetary reward conditions (i.e. HR and LR) compared to the NR condition and were modulated according to the amount of reward (HR > LR; *p_SVC_* < 0.05; Fig. 3c). Thus, these results indicate that the activity of the VM and VP as well as that of the NAc and VMPFC during the Ready period depended on the motivational state.

The Ready cue indicated that subjects should prepare for a subsequent motor response and altered their motivational state (Fig. 1a). Numerous studies have demonstrated preparatory activity in cortical and subcortical motor-related regions (Evarts and Tanji 1976; Tanji and Evarts 1976; Tanji et al. 1980; Jaeger et al. 1993; Gallivan et al. 2011, 2013; Ariani et al. 2015; Hirose et al. 2018). Consistent with these reports, cortical and subcortical motor-related regions, including the hand region in the contralateral M1, bilateral dorsal premotor cortex, SMA, and bilateral putamen, were significantly activated during the Ready period (cluster-level *p_FWE_* < 0.05 with a height threshold *p_uncorr._* < 0.001; right panels in Fig. 3a and Supplementary Table 1). These motor-related brain regions showed significantly greater activation according to the amount of anticipated monetary reward during the Ready period (HR > LR; left panels in Fig. 3c). Taken together, these data show that the mesolimbic system, including the NAc, and the mesocortical system, including cortical motor-related areas, were activated in the premovement Ready period and were modulated by motivational state.

### Preparatory brain activity related to subsequent motor performance

**Fig. 4:**
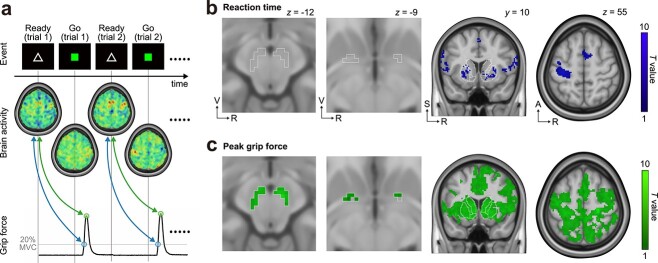
Parametric modulation of preparatory activity with subsequent motor performance. (a) Outline of parametric modulation analysis. To illustrate the brain regions where preparatory activity was correlated with subsequent motor performance, the contrasts modulated by subsequent peak grip force (green dots) and reaction time (blue dots) were estimated at the display of the “ready” cue. MVC, maximum voluntary contraction. (b) Brain regions showing preparatory activity negatively correlated with subsequent reaction time. A, anterior; R, right; S, superior; V, ventral. (c) Brain regions showing preparatory activity positively correlated with subsequent peak grip force. (b and c) The left columns show the results from small-volume correction. The regions of interest, which are outlined with white lines, indicate the ventral midbrain (left-most panel; including the bilateral ventral tegmental area, parabrachial pigmented nucleus, and substantia nigra pars compacta) and ventral pallidum (second left panel), defined according to the in vivo atlas of human subcortical brain nuclei (Pauli et al. 2018). The right columns show results from the whole-brain analysis. Outlined regions in the third column indicate the nucleus accumbens, caudate, and putamen, as defined in the in vivo atlas of human subcortical brain nuclei.

Our behavioral results showed that the trial-by-trial reaction time was not strongly correlated with peak grip force (Fig. 2c and d), whereas incentive motivation modulated both reaction time and peak grip force (Fig. 1c). In addition, the initiation and peak of force generation differed (Fig. 2b). These behavioral results suggest that distinct neural circuits control the initiation and peak of force generation. To clarify which brain regions exhibited preparatory activity correlated with subsequent motor performance, we simultaneously modeled two parametric modulation regressors corresponding to subsequent reaction time and peak grip force (Fig. 4a). Preparatory activity in cortical and subcortical motor-related regions, including the hand region of the contralateral sensorimotor cortex, SMA, and bilateral putamen, was significantly correlated with subsequent reaction time and peak grip force (cluster-level *p_FWE_* < 0.05 with a height threshold *p_uncorr._* < 0.001; right panels in Fig. 4b and c and Supplementary Table 1). Furthermore, preparatory activity in broad bilateral cortical regions, including the sensorimotor cortex, parietal cortex, middle cingulate cortex, caudate, and NAc, was also significantly correlated with subsequent peak grip force (cluster-level *p_FWE_* < 0.05 with a height threshold *p_uncorr._* < 0.001; right panels in Fig. 4c and Supplementary Table 1).

As mentioned above, shorter reaction times are considered a proxy for greater motivation (Hollerman et al. 1998; Hassani et al. 2001; Adcock et al. 2006; Mir et al. 2011). On the basis of these findings, we expected that preparatory activity in the VM and VP, modulated by the amount of anticipated monetary reward, would be related to subsequent reaction time. However, contrary to our expectations, preparatory activity in the VM and VP was significantly correlated with only subsequent peak grip force (*p_SVC_* < 0.05; left panels in Fig. 4c and Supplementary Table 2) and not reaction time (*p_SVC_* > 0.05; left panels in Fig. 4b). This result might support the weak correlation between reaction time and peak grip force (Fig. 2). To directly compare the contribution of the VM and VP to subsequent motor performance, we conducted an ROI-based analysis of the parametric modulation contrasts, which reflect the degree of association between premovement activity and subsequent motor performance (Supplementary Fig. 2). The contrast estimates for subsequent reaction time were not significant in either the VM (one-sample Wilcoxon rank-sum test; *V* = 167, *p* > 0.99) or VP (*V* = 149, *p* = 0.70). In contrast, the contrast estimates for subsequent peak grip force were significantly greater in the VM than in the VP (Wilcoxon signed-rank test; *V* = 515, *p* = 0.0087), although they were significant in both the VM (one-sample Wilcoxon rank-sum test; *V* = 364, *p* = 3.28 × 10^−6^) and VP (*V* = 291, *p* = 0.026). These results suggest that the VM is more closely associated with the peak of future force generation than the VP. Since the ready-set-go task only required participants to respond as fast as possible, they were motivated to shorten reaction time but not to facilitate peak grip force. Therefore, the current observations suggest that motivation-related VM unconsciously controls the peak of force generation associated with incentive motivation rather than the initiation of force generation directly motivated by the behavioral task.

### Movement-related brain activity

Next, we analyzed whether movement-related activity in the Go period correlated with ongoing motor performance. Broad motor-related cortical regions and bilateral reward-related striatal regions were significantly activated in the Go period (cluster-level *p_FWE_* < 0.05 with a height threshold *p_uncorr._* < 0.001; Supplementary Fig. 3a; Supplementary Table 3). These brain regions showed significantly greater activation in the Go period in the order of HR, LR, and NR (Supplementary Fig. 3b and c). Reaction time (cluster-level *p_FWE_* < 0.05 with a height threshold *p_uncorr._* < 0.001; Supplementary Fig. 3d) and peak grip force (Supplementary Fig. 3e) were associated with movement-related activity in the contralateral M1. For the striatal regions, reaction time was negatively correlated with movement-related activity mainly in the bilateral putamen (Supplementary Fig. 3d). Meanwhile, peak grip force was positively correlated with movement-related activity in the bilateral caudate nucleus and NAc (Supplementary Fig. 3e).

The bilateral VM and VP were significantly activated in the Go period (*p_SVC_* < 0.05; Supplementary Fig. 3a; Supplementary Table 4). Similar to preparatory activity (Fig. 3b and c), movement-related activities in the VM and VP were greater in the reward-anticipating conditions (i.e. HR and LR) compared to the NR condition (*p_SVC_* < 0.05; Supplementary Fig. 3b; Supplementary Table 4) and were modulated according to the amount of the expected rewards (i.e. HR > LR; Supplementary Fig. 3c). VM movement-related activity was correlated with ongoing peak grip force (*p_SVC_* < 0.05; Supplementary Fig. 3e; Supplementary Table 4) but not with reaction time (*p_SVC_* > 0.05; Supplementary Fig. 3d). On the other hand, VP movement-related activity was correlated with both reaction time (*p_SVC_* < 0.05; Supplementary Fig. 3d; Supplementary Table 4) and peak grip force (*p_SVC_* < 0.05; Supplementary Fig. 3e). Moreover, we conducted an ROI-based analysis to directly compare the association between regional movement-related activity and actual motor performance observed in the same Go period (Supplementary Fig. 2d–f). Unlike preparatory activity, there was no difference between the VM and VP in contrast estimates of either reaction time (Wilcoxon signed-rank test; *V* = 387, *p* = 0.71) or peak grip force (*V* = 364, *p* > 0.99).

## Discussion

In the present study, we manipulated the level of motivation with the expected monetary reward, which improved reaction time (Fig. 1a). According to the averages from each condition, the behavioral results demonstrated that monetary incentives improved not only reaction time but also peak grip force (Fig. 1c), implying that the mechanisms for the initiation and peak of force generation are identical. However, the correlation between reaction time and peak grip force was weak across trials (Fig. 2d). Furthermore, the timings of the initiation and peak of force generation were distinct (Fig. 2b). These behavioral findings suggest that distinct neural circuits control the initiation and peak of force generation. Indeed, our fMRI results showed that reaction time and peak grip force were correlated with premovement activity in different neural systems (Fig. 4).

Our behavioral results showed that reaction time decreased significantly with increased expected monetary rewards (Fig. 1c), a proxy for motivational state (Hollerman et al. 1998; Hassani et al. 2001; Ramnani and Miall 2003; Adcock et al. 2006; Mir et al. 2011). Similarly, premovement activity in the mesolimbic system increased with motivational incentives (Fig. 3), consistent with previous reports (Knutson et al. 2005; Tobler et al. 2005; Matsumoto and Hikosaka 2009; Takakuwa et al. 2017). However, to our knowledge, no data from either animal or human experiments have shown a relationship between activation of the VM and reaction time. Similarly, our fMRI results showed that the mesolimbic system, including the VM, was not involved in the initiation of force generation (Fig. 4b). Despite the decrease in average reaction time with increasing monetary incentive (Fig. 1c), it remains unclear why the trial-by-trial reaction time was independent of the incentive-related activity in the mesolimbic system (Fig. 4b). Further studies are needed to elucidate this mystery. Conversely, our results showed that the premovement preparatory activity in the corticostriatal network, including the M1, premotor cortex, SMA, and putamen, correlated with the subsequent reaction time (Fig. 4b). In nonhuman primate studies, the preparatory activity in neurons of the putamen (Jaeger et al. 1993) and M1 (Nashef et al. 2018) was closely linked with movement initiation. A recent magnetoencephalographic study in humans reported that reaction time can be predicted from premovement activity in the M1 as well as in the premotor cortex (Ohata et al. 2016). Furthermore, preparatory activity in the anterior striatum, including the putamen, is modulated by an upcoming reward (Hollerman et al. 1998; Hassani et al. 2001). A human neuroimaging study showed a similar interaction between reward anticipation and preparatory activity in the premotor cortex (Ramnani and Miall 2003). Thus, our present findings on the relationship between reaction time and brain activation are consistent with those of previous reports. Taken together, our results suggest that reaction time is controlled through the corticostriatal network rather than the mesocortical system.

Surprisingly, although participants were motivated to respond as quickly as possible, the level of premovement activity in the VM was correlated only with subsequent peak grip force and not reaction time (Fig. 4b). Why was premovement activity in the mesocortical system, including the VM, closely associated with the peak of subsequent force generation (Fig. 4b)? VM neurons, including DA and glutaminergic neurons, have monosynaptic projections to motor-related cortical areas in nonhuman primates and rodents (Gaspar et al. 1992; Williams and Goldman-Rakic 1998; Hosp et al. 2015; Zubair et al. 2021). The human ventral tegmental area is anatomically connected to motor-related areas, including the M1, premotor cortex, and SMA, according to diffusion tractography (Hosp et al. 2019). These motor-related cortical areas, innervated by the VM, innervate the spinal cord via the corticospinal pathways in nonhuman primates (He et al. 1993, 1995) and humans (Usuda et al. 2022). A recent study in monkeys demonstrated the existence of a multisynaptic VM–spinal pathway that generates muscle activation associated with the activation level of the VM (Suzuki et al. 2022). According to these observations, the activation of VM neurons might be functionally important for boosting force generation. Thus, it is plausible that the activity of VM neurons driven by incentive motivation unconsciously facilitates subsequent peak grip force through a VM-corticospinal pathway.

Although the peak grip force was not related to whether participants would receive monetary rewards, VM premovement activity correlated with subsequent peak grip force. This finding raises the possibility that the association between VM premovement activity and peak grip force is independent of motivational states. To investigate this possibility, we analyzed the correlation between premovement activities and subsequent motor performances only in the NR condition. Notably, we did not find any significant relationship between VM premovement activity and subsequent motor performance in the NR condition (Supplementary Fig. 4). This result supports our conclusion that premovement VM activity, which is driven by incentive motivation, is involved in the control of peak grip force. However, since the number of trials in the NR condition was limited in the present experimental design (i.e. 80 trials per subject), the lack of correlation might be due to lower statistical power. Thus, future studies should investigate the association between VM premovement activity and future peak grip force in a context without external rewards.

It remains unclear which subtype of neurons in the VM is involved in controlling the peak of future force generation. We propose that VM DA neurons are responsible. This hypothesis is supported by a previous study reporting that DA medications involuntarily enhanced the peak of force generation independently of the anticipated rewards in patients with Parkinson’s disease (Le Heron et al. 2018). However, fMRI data are unable to provide insight into the contribution of neuromodulators. The VM neurons that project to the M1 include not only DA but also glutaminergic and GABAergic neurons (Yamaguchi et al. 2007, 2013). Seventeen percent of M1-projecting neurons in the rodent VM are DA (Hosp et al. 2015). Future molecular imaging studies should investigate whether the premovement activity in the VM, which correlates with the peak of future force generation, is mediated by dopamine.

Another potential pathway is the nigrostriatal DA projections, which control the gain of the corticobasal ganglia loop (Barter et al. 2015). Indeed, premovement activity in the bilateral anterior caudate nucleus was also correlated with subsequent peak grip force (Fig. 4b). Thus, it remains possible that VM DA activity modulates striatal activity, which increases cortical motor-related activity via striothalamocortical projections.

Premovement activity in the VP, as well as the VM, correlated with the peak grip force in the present study (Fig. 4c). A previous study demonstrated that activation of the bilateral VP (representing the motivational state), M1, and SMA is correlated with grip force during movement execution (Pessiglione et al. 2007). The researchers suggested that the motivational state (represented by the VP) modulates the activity of the SMA, which in turn drives muscular contractions via the M1. However, because the anatomical connections from the VP to the motor cortices are sparse (Gaspar et al. 1992), the VP seems to have less impact on the activity of the motor cortices than the VM. On the other hand, the VP has dense DA input from the ventral tegmental area and substantia nigra pars compacta (Maslowski-Cobuzzi and Napier 1994; Root et al. 2015). Consistent with this anatomical evidence, our results showed that the correlation between subsequent peak grip force and VM premovement activity was significantly greater than that with the VP (Supplementary Fig. 2). Taken together, these findings indicate that the VM, rather than the VP, might directly modulate the peak of force generation by facilitating corticospinal pathways from motor-related cortical areas.

One limitation of the present study is the limited spatial resolution of fMRI compared to animal studies. The red nucleus (RN) and the substantia nigra pars reticulata (SNr), which are involved in motor control, are close to the midbrain dopamine region. Although reward-related activity in the SNr has been less investigated than that in the SNc, SNr GABAergic neurons are also modulated by expected rewards (Bryden et al. 2011; Rossi et al. 2013). SNr neurons have indirect cortical projections to the M1 through the thalamus and descending projections to the superior colliculus and the pontine tegmentum (Deniau et al. 2007). In the present study, the VM ROI was generated according to the MRI-based in vivo atlas of human subcortical brain nuclei (Pauli et al. 2018) and defined as the region including the SNc, parabrachial pigmental nucleus, and ventral tegmental area. Although this MRI-based atlas is defined based on higher-resolution MR images (i.e. 700 μm isotropic voxels), the VM ROI was generated at a lower spatial resolution matching the spatial resolution of functional images (i.e. 2-mm isotropic voxels). Thus, it is difficult to precisely separate VM activity from SNr activity. Future studies need to clarify the precise localization of the premovement activity in the VM by using higher-resolution fMRI in an ultra-high-field MRI scanner.

In addition to premovement activity (Fig. 3 and Fig. 4), we investigated movement-related activity (Supplementary Fig. 3). During the Go period (Supplementary Fig. 3a), more global activity and greater activity were observed in not only the cortex but also in subcortical structures, including the striatum, VM, and VP, compared to the activity observed in the Ready period (Fig. 3a). Perhaps because of the greater mean activity, the activity difference between conditions was more obvious during the Ready period (Fig. 3b and c) than during the Go period (Supplementary Fig. 3b and c). In cortical motor-related regions, performance-related modulations were more prominent during the Ready period than during the Go period (compare the data shown in Fig. 4 and Supplementary Fig. 3e and f). Since the mean activity was much greater during the Go period, it is plausible that the performance-related variability is relatively smaller compared to that in the Ready period. On the other hand, performance-related modulations in subcortical regions, including the striatum, VM, and VP, were comparable between the Ready (Fig. 4b and c) and Go periods (Supplementary Fig. 3d and e). In our behavior task participants were instructed to execute ballistic movements when the Go stimulus was displayed. Thus, participants did not need to adjust force generation during movements. Due to task requirements and slow BOLD responses, it is possible that the subcortical activation that might be involved in controlling subsequent force generation might be prolonged during the Go period. Future studies should investigate whether the VM is activated during complex movements and is involved in controlling ongoing movements.

In light of the present findings, we conclude that the mesocortical DA system linking the mesolimbic and motor systems in humans is involved in controlling peak grip force under incentive motivation. Motivation has two distinct effects on behavior: a “directing” effect, determining the current goal of behavior, and an “energizing” effect, determining the vigor of actions (Dickinson and Balleine 2002; Niv et al. 2006). In our task, vigor was reflected in both the initiation and peak of force generation, which were measured by reaction time and peak grip force, respectively (Fig. 1). Given this framework, the present findings imply that the mesocortical system mediates the “energizing” effect of motivation on force generation but is independent of the initiation of force generation. Dysfunction of the DA system is associated not only with motor deficits in Parkinson’s disease (Przedborski 2017) but also with motivational deficits (e.g. apathy) in mental disorders, such as depression (Schapira et al. 2017). Furthermore, in sports, it is plausible that athletes may drive activation of the mesocortical system, including motor-related areas, to achieve their absolute goal. The current findings shed light on the neural mechanisms of mind–motor interactions and may facilitate the development of psychophysiological therapy in the clinical and sports domains.

## Supplementary Material

20230923_SupplementaryMaterial_bhad376

## Data Availability

Original data that supports the findings of this study are available from the corresponding authors (S.K.S and Yu.Ni.) upon reasonable request.
