# Supplementary material for: Premovement activity in the mesocortical system links peak force but not initiation of force generation under incentive motivation
Source: Cereb Cortex. 2023 Oct 9;33(23):11408–19. doi: 10.1093/cercor/bhad376 (PMC10690858; doi:10.1093/cercor/bhad376)
Supplement: 20230923_SupplementaryMaterial_bhad376 [file 20230923_supplementarymaterial_bhad376.zip › 20230923_SupplementaryMaterial_bhad376.pdf]

Supplementary material

Premovement activity in the mesocortical system links peak force but not initiation of force generation under incentive motivation

Sho K. Sugawara[1,2,3,*], Tetsuya Yamamoto[2], Yoshihisa Nakayama[1], Yuki H. Hamano[2], Masaki Fukunaga[2,3], Norihiro Sadato[2,3,4], Yukio Nishimura[1,*]

[1]Neural Prosthetics Project, Tokyo Metropolitan Institute of Medical Science, Setagaya, Tokyo, 156-8506, Japan
[2]Section of Brain Function Information, National Institute for Physiological Sciences, Okazaki, Aichi, 444-8585, Japan
[3]The Graduate University for Advanced Studies, SOKENDAI, Hayama, Kanagawa, 340-0193, Japan
[4]Research Organization of Science and Technology, Ritsumeikan University, Kusatsu, Shiga, 525-8577, Japan

Corresponding author
Sho K. Sugawara, PhD (lead contact)
Neural Prosthetics Project, Tokyo Metropolitan Institute of Medical Science, Kamikitazawa 2-1-6, Setagaya, 156-8506, Tokyo, Japan
E-mail: sugawara-sh@igakuken.or.jp
Telephone: +81-3-6379-8411
Fax: +81-3-6834-2375

Yukio Nishimura, PhD
Neural Prosthetics Project, Tokyo Metropolitan Institute of Medical Science, Kamikitazawa 2-1-6, Setagaya, 156-8506, Tokyo, Japan
E-mail: nishimura-yk@igakuken.or.jp
Telephone: +81-3-6834-2373
Fax: +81-3-6834-2375

Running title
The mesocortical system mediates motivated force

Supplementary figures

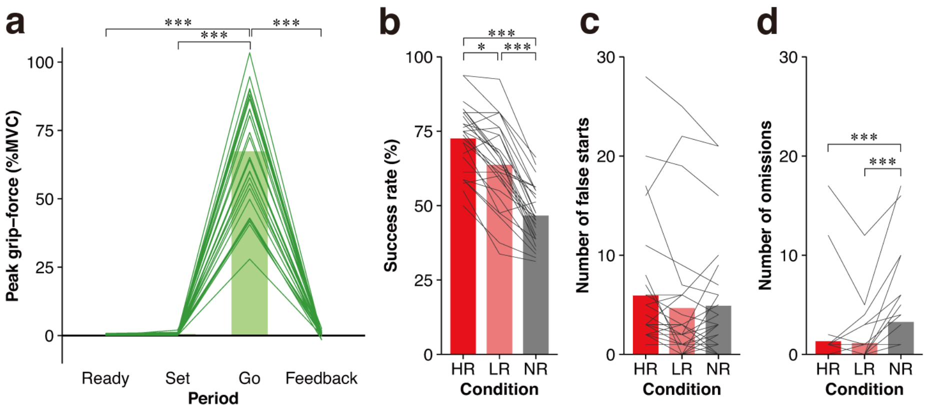

Supplementary Figure 1. Additional behavioral data. (a) Peak grip force during each task phase. Bars and lines represent the mean and individual data, respectively. (b) The percentage of successful responses was in the order of high reward (HR), low reward (LR), and no reward (NR). (c) The number of false starts did not differ among the conditions. (d) The number of omissions was significantly greater in the NR condition than in the other conditions. Bars represent the mean across subjects; lines represent the values of each participant. Colors denote three conditions: HR (red), LR (light red), and NR (gray). ***$p < 0.001$ and **$p < 0.01$ (Bonferroni corrected).

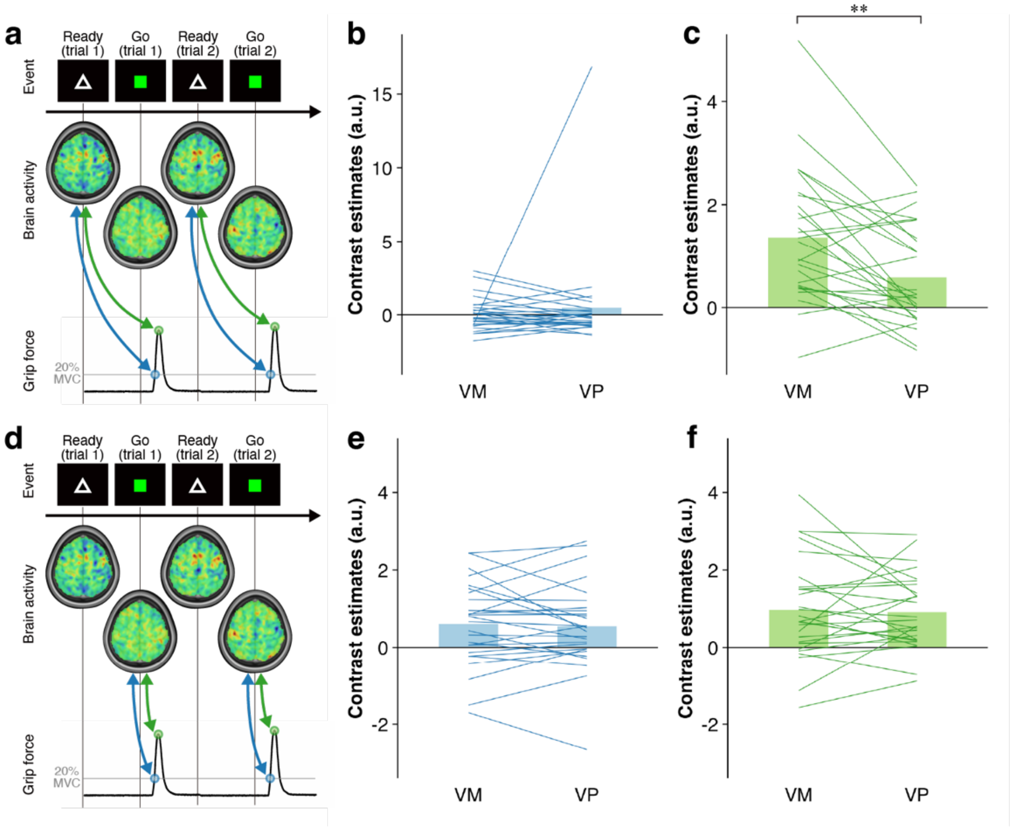

Supplementary Figure 2. Comparing contrast estimates related to parametric modulation with motor performance between the ventral midbrain (VM) and ventral pallidum (VP). To directly compare the association between regional brain activity and motor performance, we conducted an ROI-based analysis. (a) Regarding premovement activity, parametric modulation contrasts did not differ between the VM and VP in response to (b) reaction time and (c) peak grip force. (d) Regarding movement-related activity, parametric modulation contrasts did not differ between the VM and VP in either (e) reaction time or (f) peak grip force. Bars and lines represent the mean and individual data, respectively. ***p* < 0.01 (Wilcoxon signed-rank test).

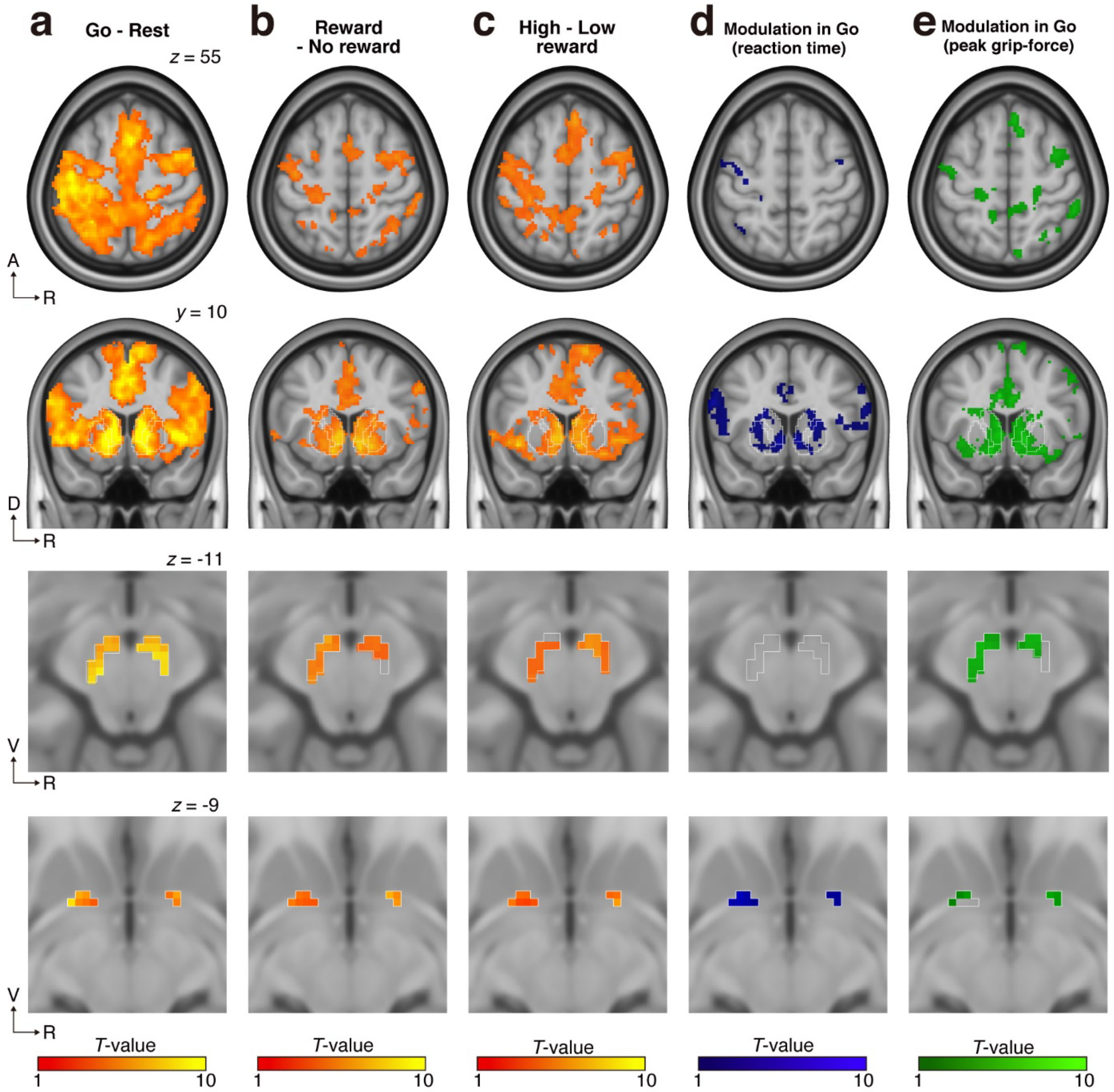

Supplementary Figure 3. Movement-related activity. (a) Mean movement-related activity across all trials. (b) Reward effect on movement-related activities (i.e., [HR + LR] > NR). (c) The effect of the amount of the expected monetary gain on movement-related activities (i.e., HR > LR). (d) Movement-related activity negatively correlated with reaction time. (e) Movement-related activity positively correlated with peak grip force. For these fMRI results, the first and second panels show the results from whole-brain analysis, and the third and fourth panels show the results from a small-volume correction in the VM and VP, respectively. Statistical thresholds were cluster-level P_{FWE} <.05 with peak-level $P_{uncorr.}$ <.001 for whole-brain analysis and peak-level P_{FWE} <.05 for small-volume correction. According to an *in vivo* atlas of human subcortical brain nuclei (Pauli et al., 2018), the VM, VP, and striatal regions are outlined with white lines.

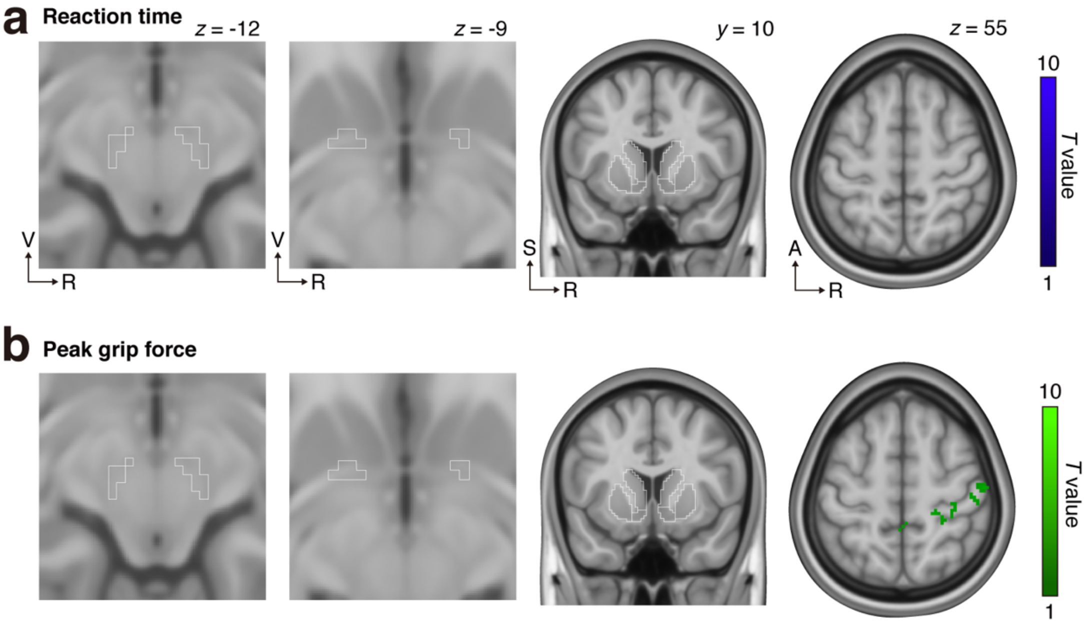

Supplementary Figure 4. Premovement activity only in the no reward condition. (a) Premovement activity negatively correlated with subsequent reaction time and (b) positively correlated with subsequent peak grip force. The left columns show the results after small-volume correction. The regions of interest, which are outlined with white lines, indicate the ventral midbrain (left-most panel) and ventral pallidum (second left panel), defined according to the *in vivo* atlas of human subcortical brain nuclei (Pauli et al. 2018). The right columns show the results of whole-brain analysis. Outlined regions in the third column indicate the nucleus accumbens, caudate, and putamen, as defined in the *in vivo* atlas of human subcortical brain nuclei.

Supplementary tables

Supplementary Table 1. Premovement activity in the whole brain (*n* = 27)

Cluster size (mm³)	Cluster p_{FWE}	MNI coordinates x	y	z	Z value	Hemi	Anatomical label
Ready – Rest							
26,728	$<1.00 \times 10^{-16}$	-3	4	58	6.13	L	Supplementary motor area
		-40	-20	56	6.34	L	Precentral gyrus
3,608	2.83×10^{-13}	40	-7	52	4.91	R	Superior frontal gyrus
		64	6	21	5.11	R	Precentral gyrus
744	5.99×10^{-4}	-34	-46	43	4.21	L	Inferior parietal lobule
1,904	2.65×10^{-8}	-21	-61	41	4.79	L	Middle occipital gyrus
432	1.96×10^{-2}	-53	2	39	4.32	L	Precentral gyrus
952	7.66×10^{-5}	34	45	28	3.73	R	Middle frontal gyrus
1,136	1.40×10^{-5}	-32	52	21	4.07	L	Middle frontal gyrus
1,400	1.43×10^{-6}	49	6	4	5.58	R	Insula
11,896	$<1.00 \times 10^{-16}$	-21	13	6	6.46	L	Putamen
		-49	4	4	5.79	L	Insula
7,248	$<1.00 \times 10^{-16}$	23	6	4	6.37	R	Putamen
11,600	$<1.00 \times 10^{-16}$	21	-104	4	5.97	R	Superior occipital gyrus
		31	-96	-3	5.42	R	Inferior occipital gyrus
3,648	2.22×10^{-13}	-5	-17	0	5.02	L	Thalamus
11,200	$<1.00 \times 10^{-16}$	-21	-102	0	6.32	L	Middle occipital gyrus
		-19	-100	-11	5.8	L	Inferior occipital gyrus
3,536	4.40×10^{-13}	14	-50	-20	5.96	R	Cerebellum IV/V

		36	-50	-29	4.92	R	Cerebellum VI
616	2.34×10^{-3}	-29	-54	-29	4.37	L	Cerebellum VI
Reward – No reward							
282,952	$<1.00 \times 10^{-16}$	-40	-15	54	6.22	L	Precentral gyrus
		1	15	50	6.15	R	Supplementary motor area
		23	4	8	6.48	R	Putamen
		-14	-17	6	6.03	L	Thalamus
		-10	11	0	6.17	L	Caudate
		-23	9	0	6.41	L	Putamen
		38	19	-3	5.92	R	Insula
		12	13	-3	6.03	R	Caudate
		10	-50	-13	5.89	R	Cerebellum IV/V
		-1	-48	-22	6.05	L	Vermis III
		44	-52	-33	5.91	R	Cerebellum crus I
2,432	4.45×10^{-9}	-29	43	28	5.07	L	Middle frontal gyrus
2,096	3.97×10^{-8}	38	50	26	4.84	R	Middle frontal gyrus
808	7.76×10^{-4}	49	-52	-3	3.92	R	Middle temporal gyrus
		55	-50	-13	4.31	R	Inferior temporal gyrus
1,840	2.28×10^{-7}	-53	-61	-5	4.92	L	Middle temporal gyrus
		-36	-63	-5	4.26	L	Fusiform gyrus
		-53	-72	-7	3.82	L	Inferior occipital gyrus
14,888	$<1.00 \times 10^{-16}$	18	-96	-5	5.36	R	Calcarine gyrus
		-32	-89	-7	4.8	L	Middle occipital gyrus
		-19	-93	-7	5.89	L	Inferior occipital gyrus
High reward – Low reward							
527,656	$<1.00 \times 10^{-16}$	8	-31	43	6.42	R	Caudal cingulate zone
		5	26	32	6.54	R	Anterior portion of rostral cingulate zone

		14	2	15	6.9	R	Caudate
		49	19	6	6.38	R	Inferior frontal gyrus
		38	6	4	6.57	R	Insula
		-51	4	4	6.52	L	Inferior frontal gyrus
		-19	-98	2	6.67	L	Middle occipital gyrus
		38	-91	0	6.4	R	Middle occipital gyrus
		36	2	-5	6.52	R	Putamen
		-27	-85	-16	6.7	L	Lingual gyrus
352	0.048	-23	52	-13	3.96	L	Anterior orbitofrontal gyrus
384	0.032	49	0	-37	4.1	R	Inferior temporal gyrus
Parametric modulation with peak grip force							
459,984	$<1.00 \times 10^{-16}$	-8	2	67	5.95	L	Supplementary motor area
		25	-24	56	6.3	R	Precentral gyrus
		8	24	30	5.66	R	Anterior portion of rostral cingulate zone
		-27	-83	23	5.94	L	Middle occipital gyrus
		34	-26	21	5.89	R	Insula
		18	-20	13	5.95	R	Thalamus
		-14	-13	8	5.67	L	Thalamus
		21	19	0	5.82	R	Caudate
		27	22	-5	5.9	R	Putamen
		-14	-4	-5	6.04	L	Globus pallidus
		-10	-96	-7	5.91	L	Calcarine gyrus
		1	-70	-35	5.85	R	Vermis VIII
Parametric modulation with reaction time							
4,448	4.15×10^{-14}	-25	-28	65	4.91	L	Postcentral gyrus
		-34	-28	60	5.5	L	Precentral gyrus
920	2.76×10^{-4}	1	2	54	4.52	R	Supplementary motor area
696	2.20×10^{-3}	-49	2	30	3.8	L	Precentral gyrus

1,360	7.38×10^{-6}	42	13	8	4.53	R	Inferior frontal gyrus
		47	6	6	4.73	R	Insula
3,520	6.31×10^{-12}	14	2	13	4.77	R	Caudate
		25	4	6	5.3	R	Putamen
		23	-4	6	4.95	R	Globus pallidus
3,736	1.89×10^{-12}	-29	-15	8	4.98	L	Putamen
		-23	2	2	4.98	L	Globus pallidus
1,168	3.39×10^{-5}	-51	6	6	4.7	L	Inferior frontal gyrus
6,824	$<1.00 \times 10^{-16}$	14	-52	-16	5.25	R	Cerebellum IV/V
		1	-63	-22	5.06	R	Vermis VI
		31	-59	-24	5.21	R	Cerebellum VI
1,008	1.29×10^{-4}	-23	-63	-22	4.97	L	Cerebellum VI
		-45	-54	-33	4.6	L	Cerebellum crus I

Hemi, hemisphere; L, left; MNI, Montreal Neurological Institute; R, right.

Supplementary Table 2. Premovement activity in the ventral pallidum and ventral midbrain (n = 27)

Cluster size (mm³)	p_{SVC}	MNI coordinates			Z value	Hemi	Anatomical label
		x	y	z			
Ready – Rest							
80	5.52×10^{-3}	-5	-17	-13	3.76	L	Parabrachial pigmented area (A10)
Reward – No reward							
296	4.30×10^{-7}	-5	-13	-9	5.72	L	Parabrachial pigmented area (A10)
48	2.74×10^{-5}	-14	4	-9	4.55	L	Ventral pallidum
32	1.13×10^{-6}	14	2	-9	5.18	R	Ventral pallidum
312	1.80×10^{-6}	5	-17	-13	5.47	R	Parabrachial pigmented area (A10)
	5.65×10^{-4}	8	-24	-16	4.34	R	Substantial nigra pars compacta (A9)
High reward – Low reward							
48	1.78×10^{-5}	-14	4	-9	5.86	L	Ventral pallidum
32	1.43×10^{-5}	14	2	-9	5.74	R	Ventral pallidum
320	1.30×10^{-6}	5	-15	-13	5.53	R	Parabrachial pigmented area (A10)
296	1.64×10^{-5}	-8	-24	-13	5.06	L	Substantial nigra pars compacta (A9)
	2.49×10^{-5}	-5	-13	-9	4.98	L	Parabrachial pigmented area (A10)

Parametric modulation with peak grip force

40	1.20×10^{-3}	-16	2	-9	3.67	L	Ventral pallidum
24	1.10×10^{-3}	12	4	-9	3.69	R	Ventral pallidum
304	1.10×10^{-6}	8	-17	-11	5.56	R	Parabrachial pigmented area (A10)
	1.50×10^{-3}	10	-24	-13	4.11	R	Substantial nigra pars compacta (A9)
248	1.41×10^{-4}	-5	-13	-11	4.64	L	Parabrachial pigmented area (A10)

Parametric modulation with reaction time

	No significant voxels

Hemi, hemisphere; L, left; MNI, Montreal Neurological Institute; R, right.

Supplementary Table 3. Movement-related activity in the whole brain ($N = 27$)

Cluster size (mm³)	Cluster P_{FWE}	MNI coordinates x	y	z	Z value	Hemi	Anatomical label
Movement – Rest							
675624	$<1.00\times10^{-16}$	16	-11	10	7.67	R	Thalamus
		-14	-17	10	Inf	L	Thalamus
		-10	6	6	7.78	L	Caudate
		10	9	4	7.76	R	Caudate
		42	11	0	Inf	R	Insula
		3	-57	-9	Inf	R	Vermis IV/V
		12	-52	-16	Inf	R	Cerebellum IV/V
		23	-57	-22	Inf	R	Cerebellum VI
		1	-63	-22	Inf	R	Vermis VI
984	3.95×10^{-5}	51	4	-31	4.85	R	Middle temporal gyrus
		40	6	-33	3.14	R	Temporal pole
		40	2	-42	4.18	R	Inferior temporal gyrus
Reward - No reward							
2608	1.65×10^{-9}	23	-26	71	4.35	R	Precentral gyrus
1144	4.54×10^{-5}	-25	-52	58	4.14	L	Superior parietal lobule
		-32	-48	50	4.21	L	Inferior parietal lobule
408	4.57×10^{-2}	-3	-11	50	4.22	L	Supplementary motor area
9104	$<1.00\times10^{-16}$	34	-54	50	4.71	R	Inferior parietal lobule
2704	9.17×10^{-10}	-21	-72	41	4.21	L	Superior parietal lobule
		-16	-65	39	4.34	L	Superior occipital gyrus
		-27	-67	28	4.18	L	Middle occipital gyrus
600	5.96×10^{-3}	34	-76	34	3.71	R	Middle occipital gyrus
1208	2.71×10^{-5}	-45	-41	26	4.3	L	Supramarginal gyrus
544	1.06×10^{-2}	57	-31	23	3.48	R	Supramarginal gyrus
752	1.37×10^{-3}	34	35	21	4.75	R	Middle frontal gyrus

1056	9.73×10^{-5}	57	6	21	3.87	R	Precentral gyrus
		49	4	21	4.08	R	Inferior frontal gyrus
1016	1.31×10^{-4}	-62	9	6	4.03	L	Inferior frontal gyrus
		-45	4	4	4.18	L	Insula
		-55	6	0	4.96	L	Temporal pole
178104	$<1.00\times10^{-16}$	36	19	0	5.83	R	Insula
		10	17	-3	6.62	R	Caudate
		-8	11	-5	5.88	L	Caudate
		5	-57	-11	6.12	R	Vermis IV/V
		1	-48	-22	6.11	R	Vermis III
		3	-57	-24	6.32	R	Vermis VI
		1	-61	-35	5.78	R	Vermis IX
		1	-67	-35	5.82	R	Vermis VIII
1000	1.50×10^{-4}	53	-44	-11	4.51	R	Inferior temporal gyrus
1232	2.24×10^{-5}	18	35	-22	4.3	R	Medial orbitofrontal gyrus
High reward - Low reward							
358808	$<1.00\times10^{-16}$	16	15	65	6.09	R	Supplementary motor area
		12	-17	17	5.98	R	Thalamus
		-23	-96	6	6.33	L	Middle occipital gyrus
		10	17	4	5.83	R	Caudate
		29	-93	4	6.31	R	Middle occipital gyrus
		-38	11	-7	6.02	L	Insula
		27	22	-11	6.78	R	Insula
464	1.56×10^{-2}	47	-41	60	3.4	R	Superior parietal lobule
1160	1.56×10^{-5}	27	-59	56	3.87	R	Superior parietal lobule
6856	$<1.00\times10^{-16}$	38	-54	39	3.63	R	Angular gyrus
		62	-44	43	4.78	R	Supramarginal gyrus
520	8.19×10^{-3}	53	-22	30	3.56	R	Postcentral gyrus
		57	-28	26	3.2	R	Supramarginal gyrus

Parametric modulation with reaction time

520	8.49×10^{-3}	3	-4	71	3.7	R	Supplementary motor area
		-5	0	63	3.62	L	Supplementary motor area
928	1.34×10^{-4}	-12	-17	69	4.12	L	Paracentral lobule
		-25	-28	67	3.32	L	Postcentral gyrus
		-19	-22	63	3.51	L	Precentral gyrus
552	5.94×10^{-3}	-34	-52	52	3.56	L	Inferior parietal lobule
2736	1.50×10^{-10}	-51	-35	50	4.27	L	Inferior parietal lobule
		-55	-26	30	4.64	L	Supramarginal gyrus
		-60	-15	19	4.43	L	Precentral gyrus
21448	$<1.00\times10^{-16}$	-53	0	43	4.79	L	Precentral gyrus
		-16	-13	19	5.57	L	Caudate
		-27	-17	10	5.15	L	Putamen
		-49	6	8	5.14	L	Inferior frontal gyrus
		-42	0	2	5.44	L	Insula
616	2.97×10^{-3}	-8	13	37	3.49	L	Anterior portion of rostral cingulate zone
		3	9	34	3.93	R	Middle cingulate cortex (23ab)
		-1	11	28	3.24	L	Anterior cingulate cortex
536	7.10×10^{-3}	1	-4	30	4.7	R	Middle cingulate cortex (23ab)
920	1.44×10^{-4}	60	-17	26	4.09	R	Supramarginal gyrus
704	1.18×10^{-3}	-32	48	17	3.86	L	Middle frontal gyrus
		-32	41	10	4.1	L	Inferior frontal gyrus
17296	$<1.00\times10^{-16}$	29	2	13	4.89	R	Putamen
		38	2	10	5.14	R	Insula
		8	13	-3	5.31	R	Caudate
44296	$<1.00\times10^{-16}$	23	-87	-3	5	R	Lingual gyrus
		18	-93	-3	4.93	R	Calcarine gyrus
		1	-61	-5	5.17	R	Vermis IV/V

		-16	-96	-7	5.41	L	Calcarine gyrus
		3	-74	-9	5.2	R	Vermis VI
		8	-65	-13	5.08	R	Cerebellum VI
		-1	-67	-20	5.18	L	Vermis VI
		-1	-54	-22	5.47	L	Vermis IV/V
		-29	-63	-24	5.18	L	Cerebellum VI
		47	-70	-29	4.94	R	Cerebellum crus I
		-5	-70	-29	5.54	L	Cerebellum crus I
		1	-65	-33	5.11	R	Vermis VIII
		23	-70	-48	5.24	R	Cerebellum VIII
552	5.94×10^{-3}	16	52	-9	4.21	L	Superior orbitofrontal gyrus
		21	43	-18	3.98	L	Medial orbitofrontal gyrus
632	2.50×10^{-3}	-25	50	-16	4.01	L	Anterior orbitofrontal gyrus
		-19	41	-16	4.92	L	Medial orbitofrontal gyrus
408	3.13×10^{-2}	25	-37	-44	4.07	L	Cerebellum X
		36	-37	-33	3.43	L	Cerebellum VI
1160	1.67×10^{-5}	-19	-70	-55	4.95	L	Cerebellum VIII
Parametric modulation with peak grip force							
3848	9.23×10^{-13}	10	4	69	5.07	R	Supplementary motor area
		-8	-2	76	4.87	L	Supplementary motor area
2296	9.40×10^{-9}	27	-26	65	3.79	R	Precentral gyrus
4224	1.21×10^{-13}	38	4	60	4.58	R	Middle frontal gyrus
		49	2	45	4.46	R	Precentral gyrus
3192	3.78×10^{-11}	-47	-7	58	4.26	L	Precentral gyrus
		-45	-13	50	4.44	L	Postcentral gyrus
2000	6.77×10^{-8}	51	-41	56	4.35	R	Inferior parietal lobule
		55	-39	32	4.27	R	Supramarginal gyrus
212472	$<1.00\times10^{-16}$	36	-57	41	5.27	R	Angular gyrus
		-5	11	32	5.44	L	Posterior portion of rostral cingulate zone

		3	-11	32	5.29	R	Middle cingulate cortex (23ab)
		-5	32	19	5.75	L	Anterior cingulate cortex
		3	43	15	5.25	R	Anterior cingulate cortex
		10	17	0	5.45	R	Caudate
		3	-61	-24	5.27	R	Vermis VI
		3	-70	-29	6.21	R	Vermis VII
		-12	-76	-35	5.52	L	Cerebellum crus II
		25	-35	-44	5.37	R	Cerebellum X
424	3.59×10^{-2}	34	22	30	3.9	R	Inferior frontal gyrus
2616	1.22×10^{-9}	-45	-37	28	4.73	L	Supramarginal gyrus
712	1.81×10^{-3}	44	-37	21	3.38	R	Supramarginal gyrus
1976	7.98×10^{-8}	21	67	2	4.59	R	Superior frontal gyrus
440	3.00×10^{-2}	14	-59	-59	4.01	R	Cerebellum VIII

Hemi, hemisphere; L, left; MNI, Montreal Neurological Institute; R, right.

Supplementary Table 4. Movement-related ventral midbrain activity ($N = 27$)

Cluster size (mm^3)	P_{SVC}	MNI coordinates x	y	z	Z value	Hemi	Anatomical label
Movement – Rest							
320	3.39×10^{-10}	-8	-22	-13	6.83	L	Parabrachial pigmented area (A10)
320	1.27×10^{-9}	10	-17	-9	6.64	R	Parabrachial pigmented area (A10)
Reward - No reward							
288	2.06×10^{-5}	-1	-17	-13	5.02	L	Parabrachial pigmented area (A10)
	2.19×10^{-4}	-8	-24	-13	4.55	L	Substantial nigra pars compacta (A9)
184	4.68×10^{-5}	5	-17	-16	4.86	R	Substantial nigra pars compacta (A9)
High reward - Low reward							
312	2.02×10^{-4}	5	-15	-11	4.56	R	Parabrachial pigmented area (A10)
232	5.64×10^{-4}	-10	-15	-9	4.34	L	Parabrachial pigmented area (A10)
Parametric modulation with reaction time							
	No significant voxels						
Parametric modulation with peak grip-force							
240	2.02×10^{-4}	-8	-15	-11	4.56	L	Substantial nigra pars compacta (A9)
	3.91×10^{-4}	-8	-20	-13	4.42	L	Parabrachial pigmented area (A10)
168	6.35×10^{-4}	8	-13	-11	4.31	R	Substantial nigra pars compacta (A9)
	1.14×10^{-3}	3	-17	-13	4.17	R	Parabrachial pigmented area (A10)

Hemi, hemisphere; L, left; MNI, Montreal Neurological Institute; R, right.
